# Fifteen into Three Does Go: Morphology, Genetics and Genitalia Confirm Taxonomic Inflation of New Zealand Beetles (Chrysomelidae: *Eucolaspis*)

**DOI:** 10.1371/journal.pone.0143258

**Published:** 2015-11-23

**Authors:** Prasad R. C. Doddala, Maria A. Minor, David J. Rogers, Steven A. Trewick

**Affiliations:** 1 Ecology Group, Institute of Agriculture and Environment, Massey University, Palmerston North, New Zealand; 2 Plant and Food Research Ltd., Havelock North, New Zealand; Inha University, REPUBLIC OF KOREA

## Abstract

*Eucolaspis* Sharp 1886 is a New Zealand native leaf beetle genus (Coleoptera: Chrysomelidae: Eumolpinae) with poorly described species and a complex taxonomy. Many economically important fruit crops are severely damaged by these beetles. Uncertain species taxonomy of *Eucolaspis* is leaving any biological research, as well as pest management, tenuous. We used morphometrics, mitochondrial DNA and male genitalia to study phylogenetic and geographic diversity of *Eucolaspis* in New Zealand. Freshly collected beetles from several locations across their distribution range, as well as identified voucher specimens from major museum collections were examined to test the current classification. We also considered phylogenetic relationships among New Zealand and global Eumolpinae (Coleoptera: Chyrosomelidae). We demonstrate that most of the morphological information used previously to define New Zealand *Eucolaspis* species is insufficient. At the same time, we show that a combination of morphological and genetic evidence supports the existence of just 3 mainland *Eucolaspis* lineages (putative species), and not 5 or 15, as previously reported. In addition, there may be another closely related lineage (putative species) on an offshore location (Three Kings Islands, NZ). The cladistic structure among the lineages, conferred through mitochondrial DNA data, was well supported by differences in male genitalia. We found that only a single species (lineage) infests fruit orchards in Hawke’s Bay region of New Zealand. Species-host plant associations vary among different regions.

## Introduction

It has been estimated that about 86% of extant species on Earth are yet to be described even after 250 years of taxonomic classification [1], creating impediment to many areas of biological research. It is also increasingly apparent that adherence to strictly defined species concepts is incompatible with evolutionary biology [2]. Pragmatic approaches focus on separately evolving metapopulation lineages, so that species conceptualization can be separated from the methodological problem of species delimitation [2–4]. Wherever possible, integrating molecular, morphological, ecological, behavioural and other related traits is now encouraged, as this supports any taxonomic decision more robustly than when any single data set is used [5]. An iterative approach to taxonomy [6] accommodates multiple data sets; with this in mind we examined the degree of morphological, genetic and geographical variation within a native genus of leaf beetles, *Eucolaspis* in New Zealand.

The leaf beetle genus *Eucolaspis* (Coleoptera: Chrysomelidae: Eumolpinae) was established by Sharp [7] for species described earlier by Fabricius [8], White [9] and Broun [10]. Fabricius [8] and White [9] each described one species, while Broun [10–13] described 13 species under the genus. Broun’s descriptions are brief and it is not possible to compile a diagnostic key from them [14]. Shaw [15] revised the New Zealand *Eucolaspis* and placed a total of five species in the genus. Subsequently, Kuschel [16] recommended that the synonymy suggested by Shaw [15] be ignored, although the justification for this was never published. Bryant and Gressitt [17] described two more species of *Eucolaspis* outside of New Zealand—*E*. *castanea* and *E*. *saltator* from Fiji. Close relatives of *Eucolaspis* might include Pacific *Colaspoides* Laporte 1833 (New Caledonia, Norfolk Island and other Pacific islands) and *Dematochroma* Baly 1864 (New Caledonia). Genus *Eucolaspis* Sharp 1886 appears to be much more diverse and widely distributed than the other three native genera of subfamily Eumolpinae in New Zealand (*Atrichatus*, *Pilacolaspis* and *Peniticus*).

Attention was recently drawn to *Eucolaspis* in New Zealand due to their status as a serious pest of apple and other fruit crops. Adult beetles feed on leaves, flowers and fruits of host plants, while larvae live underground and feed on small roots [14]. Leaf damage usually does not result in noticeable economic loss, but fruit damage results in unmarketable fruit and heavy economic loss, especially in organic horticulture [18]. Annual loss to organic apple orchards in New Zealand due to *Eucolaspis* infestation is estimated to reach 10–15 million NZD [19]. The beetles also naturally use a range of native tree and shrub species as hosts (S1 Table) [20].

The most frequently cited formal species name of the genus *Eucolaspis* in New Zealand is *E*. *brunnea* Fabricius 1781 and this species is commonly referred to as the “bronze beetle” [21]. The name “bronze beetle” has also been applied more generally to all New Zealand *Eucolaspis*. Damage in apple orchards was attributed to *E*. *brunnea* until 2007 [14,18], when *Eucolaspis* from an organic apple orchard in Hawke’s Bay were provisionally identified as *E*. *pallidipennis* [19]. However, due to the extent of variation in size and colour within beetle populations and ambiguity in the existing taxonomy, a treatment as uncertain identity was favoured [19] and the consensus has been to treat the taxonomy of *Eucolaspis* as unresolved (e.g., [19,22]).

Unresolved taxonomy diminishes research on all aspects of insect biology, including the use of targeted methods of pest control, such as the use of biological control. We explored diversity in the genus *Eucolaspis* Sharp 1886 to answer specific questions: How many species/lineages of *Eucolaspis* exist in New Zealand, and what are their geographic associations? How do these lineages relate to each other and to other New Zealand and international Eumolpinae genera? How many lineages of *Eucolaspis* infest apples in Hawke’s Bay, New Zealand?

## Materials and Methods

No specific permissions were required for the locations sampled in the study, except for private orchards, for which permissions have been obtained from the owners. All other locations are unrestricted free public access localities; the species sampled are not endangered or protected.

### Insects

**Fig 1:**
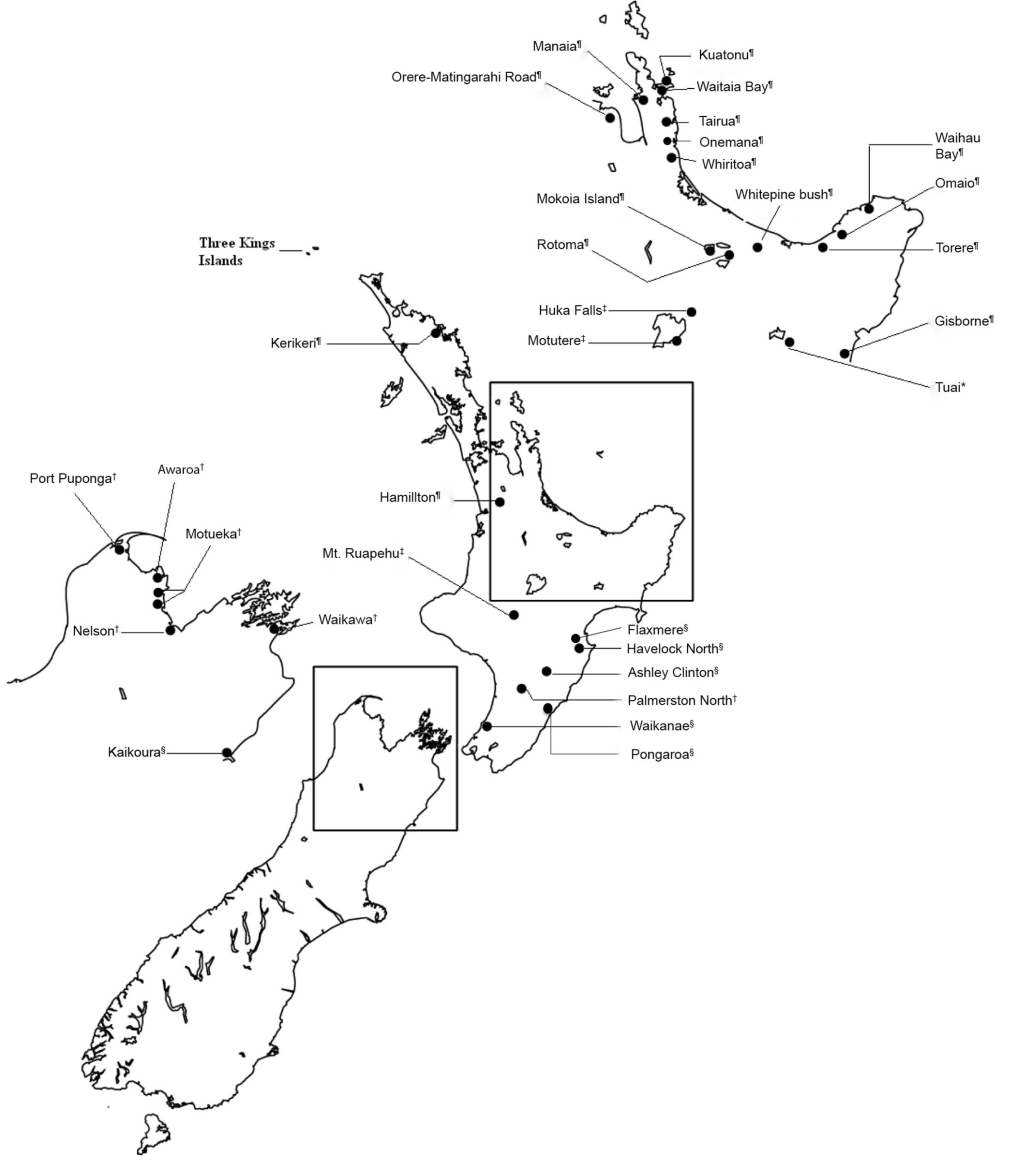
Collection localities of New Zealand *Eucolaspis* beetles used in this study. Closed circles represent sampling locations. Inset maps show details of more intensely sampled regions. Relevant localities and regions are indicated. Sampled ecological regions include ^¶^Northern North Island, ^‡^Central Volcanic Plateau, *Axial Ranges, ^†^Windward Districts and ^§^Leeward Districts.

Adult *Eucolaspis* beetles were collected from various locations and host plants throughout New Zealand (Fig 1 and S2 Table) and preserved in 95% ethanol. In addition, we examined representative named *Eucolaspis* specimens in the New Zealand Arthropod Collection (NZAC, Landcare Research Ltd., Auckland) including available type material. Specimens of related taxa, *Atrichatus ochraceus*, *A*. *aenicollis*, *Peniticus* sp. and *Pilacolaspis* sp. in the Entomology Research Museum at Lincoln University, Lincoln (LUNZ) were also examined.

### External morphology

The main characters Broun [10–13] used to delineate species were body size, body colour, pronotum shape, pronotum size, density of pronotal punctures, and density of elytral punctures. Shaw [15], whose study was based almost entirely on reexamination of Broun’s specimens at the British Museum of Natural History (BMNH), primarily used external shape and pronotal puncture density. However, neither Broun nor Shaw quantified the variation in morphology within and between putative species, but rather used relative estimates to indicate variation. It is clear that some of the characters, such as body colour, shape and size of pronotal punctures can vary greatly within populations (P.Doddala, pers. obs.).

**Fig 2:**
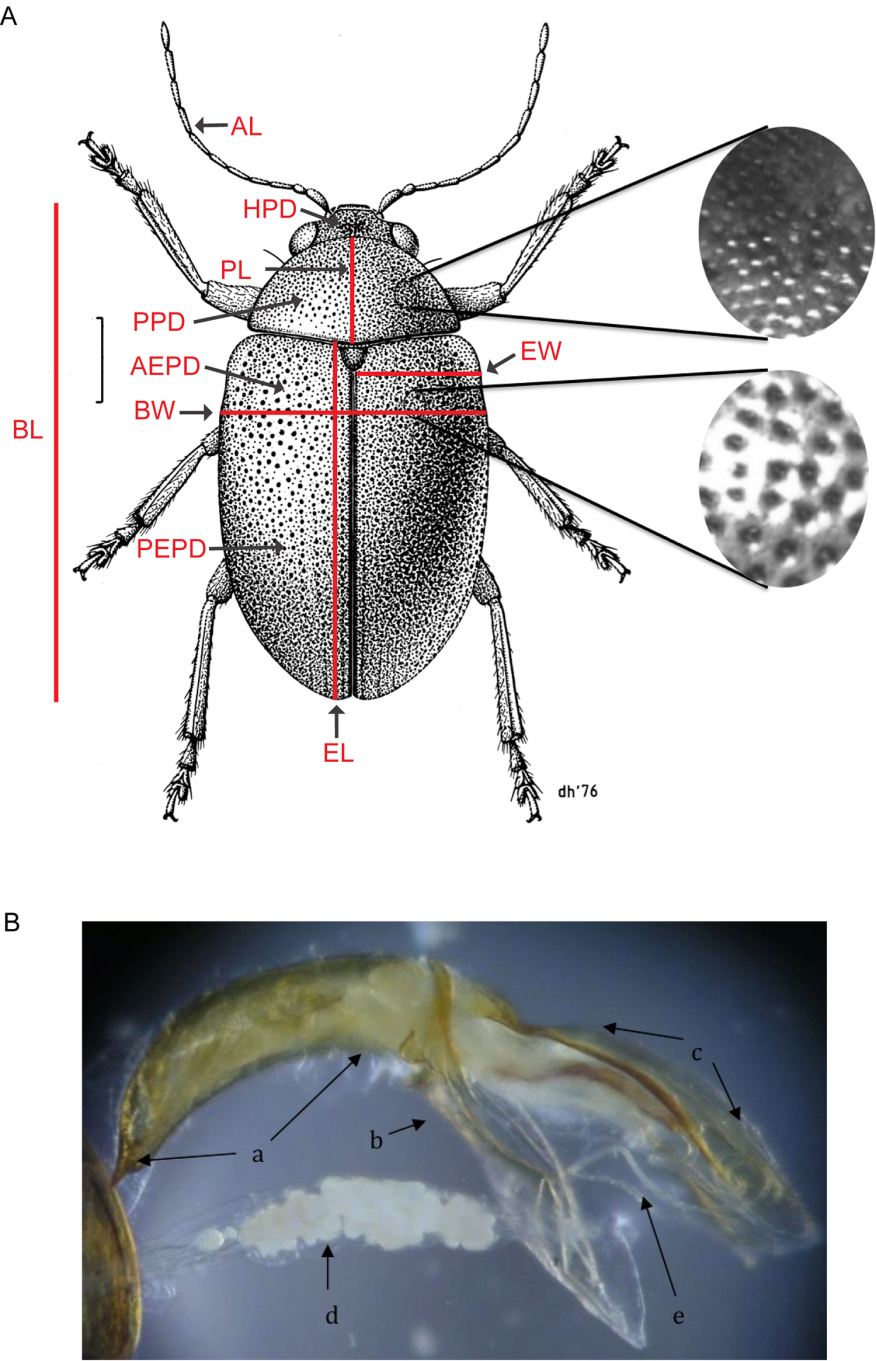
External and internal morphological characters of *Eucolaspis* assessed. (A) *Eucolaspis* (Coleoptera: Chrysomelidae) (modified from Des Helomore’s drawing of *Eucolaspis brunnea* (Fabricius)). Scale bar = 1mm. Insets show puncturation on pronotum (top) and elytra (below). Insets are not to scale. Morphometric measurements recorded are labeled: BL- body length (mm); BW- body width (mm); AL- antenna length(mm); EL- eltyra length (mm); EW- elytra width (mm); PL- pronotum length (mm); HPD- puncture density on the head (mm^-2^); PPD- puncture density on the pronotum (mm^-2^); AEPD- punctures density on anterior half of the elytra (mm^-2^); PEPD- punctures density on posterior half of the elytra (mm^-2^). (B) Aedeagus from a male *Eucolaspis* beetle collected on apple at Havelock North, New Zealand: a–aedeagus proper; b–tegmen; c–basal hood; d–ejaculatory sac; e–median ejaculatory duct.

In this study, we quantified a set of the external morphological characters previously used (Fig 2A). External morphological characters were recorded from randomly selected individuals from each locality / sample using a digital camera (Moticam 2000 2.0 MP USB 2.0; Motic Group Co., Ltd.) fitted to a dissecting microscope (Zeiss Stemi 2000-c; Carl Zeiss, Inc.). Motic Images Plus v.2.0 (Motic Group Co., Ltd.) was used to record measurements from those images.

### Morphology of male genitalia

The morphology (structure, shape and size) of internal genitalia was studied in a subset of randomly selected male beetles among fresh specimens used for external morphology. Male beetles were soaked in cold 10% potassium hydroxide for 12 hours, rinsed thoroughly in 70% ethanol followed by rinsing in dH_2_O, and then soaked for an hour in hydrogen peroxide [23]. The clearing procedure was repeated as necessary, and the cleared genitalia (Fig 2B) examined under a dissecting microscope with measurements taken using Motic Images Plus software.

### DNA extraction

Total genomic DNA was extracted from selected beetles using either a salting-out extraction method [24] with excised legs or the QIAGEN DNeasy blood and tissue kit (QIAGEN N.V.) with whole body samples. Extraction of DNA from dry museum specimens was carried out in a dedicated Ancient DNA laboratory at Massey University, Palmerston North. DNA extractions were checked for quantity and quality by gel-electrophoresis and spectrophotometry (NanoDrop; Thermo Fisher Scientific Inc.). One percent agarose gels with SYBR Safe DNA gel stain (Life Technologies Corp.) in TAE buffer (Tris-HCl, glacial acetic acid, EDTA and H_2_O) were used for electrophoreses.

### DNA amplification and sequencing

A ~700 base pairs (bp) fragment of the mitochondrial DNA Cytochrome Oxidase I (COI) locus was amplified by polymerase chain reaction (PCR) using universal insect primers, LCO1490: 5’-GGTCAACAAATCATAAACATATTGG-3’ and HCO2198: 5’-TAAACTTCAGGGTGACCAAAAAATCA-3’ [25]. A smaller but complimentary fragment (~350 bp length) was targeted in DNA templates from some of the museum specimens, using specially designed primers, BBCO1F (5’-TGACTRCTRCCCCCGTCATT-3’) and BBCO1R (5’-GGRTCWCCWCCTCCKGCAGGRTC-3’). PCR primers were designed in Geneious Pro v.5.5 (Biomatters Ltd., Auckland) using alignments of the COI nucleotide sequences obtained from modern specimens.

A 10μL reaction protocol was employed for PCR comprising of 3.3μL of Milli-Q H_2_O, 1.0μL of dNTPs mix (0.2 μL of each 2mM dNTP), 1.0μL of 10x PCR buffer, 0.8μL of 25mM MgCl_2_, 0.4μL of each 10μM primer, 2.0μL of betaine, 0.1μL *Taq* DNA polymerase enzyme (500U) (F. Hoffmann-La Roche Ltd.) and 1.0μL of the extracted DNA. Reactions were carried out in a Biometra T3000 thermocycler (Biometra GmbH) using the conditions: 94°C for 2 minutes (initial denaturation), 40 cycles (50 cycles for some of the degraded ancient DNA samples) of 94°C for 30 s (denaturation), 52°C for 30 s (annealing), 72°C for 1 minute 30 s (primer extension) and 72°C for 8 minutes (final extension). PCR products were purified using the SAP (Shrimp Alkaline Phosphase) / EXO1 (Exo nuclease I) digest protocol and were sequenced from the 5’ end using one of the forward primers (either LCO1490 or BBCOIF). Sequencing used Big Dye Chemistry and an ABI3730 genetic analyser (Applied Biosystems Inc.). Mitochondrial DNA sequences were obtained from a total of 117 fresh and two NZAC specimens of *Eucolaspis* and one each of *Atrichatus ochraceus*, *A*. *aenicollis* and *Peniticus* sp. (LUNZ). In addition, we obtained published COI mtDNA and 18S rDNA sequences for other Eumolpinae genera from GenBank (National Centre for Biotechnology Information–NCBI, USA) database for phylogenetic analysis of genus level relationships We also amplified and sequenced the majority of mtDNA CO1 (~1400 bp) using primers LCO1490 and TL2-N-3014 (5’-TCCAATGCACTAATCTGCCATATTA-3’) [26], and nuclear 18S rRNA using primers 18S-S22 (5’-TAATGATCCTTCCGCAGGTTCA-3’) and 18S-A1984 (5’-TCCCTGGTTGATCCTGCCAGTA-3’) [27], from representative individuals of the lineages identified.

### Data analysis

Morphometric data were tested for distributional normality using multivariate procedures. Morphometric differences between the two sexes were assessed using a t-test. Stepwise discriminant analysis was used to identify which variables (characters) contributed significantly to delineation of sample classes. Subsequently, canonical discriminant analysis was performed using the variables identified by stepwise discriminant analysis, to verify similarity / diversity of samples grouped according to ecological region, host plant, genetic lineage or genitalia shape. Sample locations were assigned to recognised New Zealand ecological regions to test for association of taxa and environment [28]. The “Axial ranges” ecological region was represented by a single sample locality and so was excluded from analyses. A 95% level of confidence was used as a significance level for all the statistical analyses. All analyses were performed using SAS v.9.2 (SAS Institute, 1992).

DNA sequence chromatograms were checked using SEQUENCHER v.4.2 (Gene Codes Corp., Michigan); ambiguous base calls were corrected manually and ambiguous end regions were trimmed. Sequences were aligned using Se-Al v2.0a11. Unique haplotypes were identified and sequence divergence was measured using DnaSP v.5 [29] after the haplotypes were aligned using Clustal-W in Geneious Pro (version 5.5) (Biomatters Ltd., Auckland) [30]. Phylogenetic analyses were conducted using MEGA (version 6.0) [31] and Geneious Pro (with plugins for MrBayes [32,33] and PAUP* [34]). The optimal models of nucleotide substitution were identified using jModeltest (version 0.1.1) [35]. Evolutionary distances between lineages were calculated in MEGA6. Species delimitation analyses were conducted in Geneious Pro using the species delimitation plugin [36], which calculates geneological concordance [2].

## Results

### Morphometric analysis

#### Morphological variation in fresh samples

Body length (BL) in fresh beetles varied from 2.69 mm to 4.45 mm (mean 3.56 mm) whereas body width (BW) varied from 1.54 mm to 3.16 mm (mean 2.14 mm) (n = 135). Punctures were denser on the pronotum than on elytra or head in all insects. Punctures on pronotum (PPD) varied in density from 160 to 810 per mm^2^, whereas punctures on head (HPD) varied from 20 to 320 per mm^2^. Elytra were less densely punctured at 50 to 180 punctures per mm^2^. There was noticeable sexual dimorphism in body shape with male beetles significantly smaller and more slender than female beetles, and having longer antennae (Table 1). There was, however, no significant difference between male and female beetles in the density of punctures on head, pronotum and elytra.

**Table 1:** Sexual dimorphism in New Zealand *Eucolaspis* beetles. Data from representative individuals among fresh beetle samples collected throughout New Zealand. BL Body length, BW body width, EL elytra length, EW elytra width, AL antennae length, PL pronotum length, HPD head puncture density, PPD pronotal puncture density, AEPD anterior elytral puncture density, PEPD posterior elytral puncture density.

Morphological character	Mean (S.E.) for ♀	Mean (S.E.) for ♂	*t*	*p*
BL (mm)	3.65 (0.04)	3.44 (0.06)	**3.16**	**.002**
BW (mm)	2.22 (0.03)	2.03 (0.04)	**3.44**	**< .001**
EL (mm)	2.88 (0.04)	2.61 (0.06)	**4.10**	**< .001**
EW(mm)	1.00 (0.02)	0.93 (0.02)	**2.79**	**.006**
AL (mm)	2.13 (0.03)	2.66 (0.06)	**-8.48**	**< .001**
PL (mm)	0.998 (0.012)	0.95 (0.02)	**2.11**	**.037**
HPD (mm^-2^)	185.7 (5.3)	171.1 (7.0)	1.69	.093
PPD (mm^-2^)	418.8 (15.4)	417.5 (22.9)	0.05	.959
AEPD (mm^-2^)	116.5 (3.4)	114.4 (4.0)	0.40	.692
PEPD (mm^-2^)	100.8 (2.5)	99.6 (2.8)	0.31	.758

**Fig 3:**
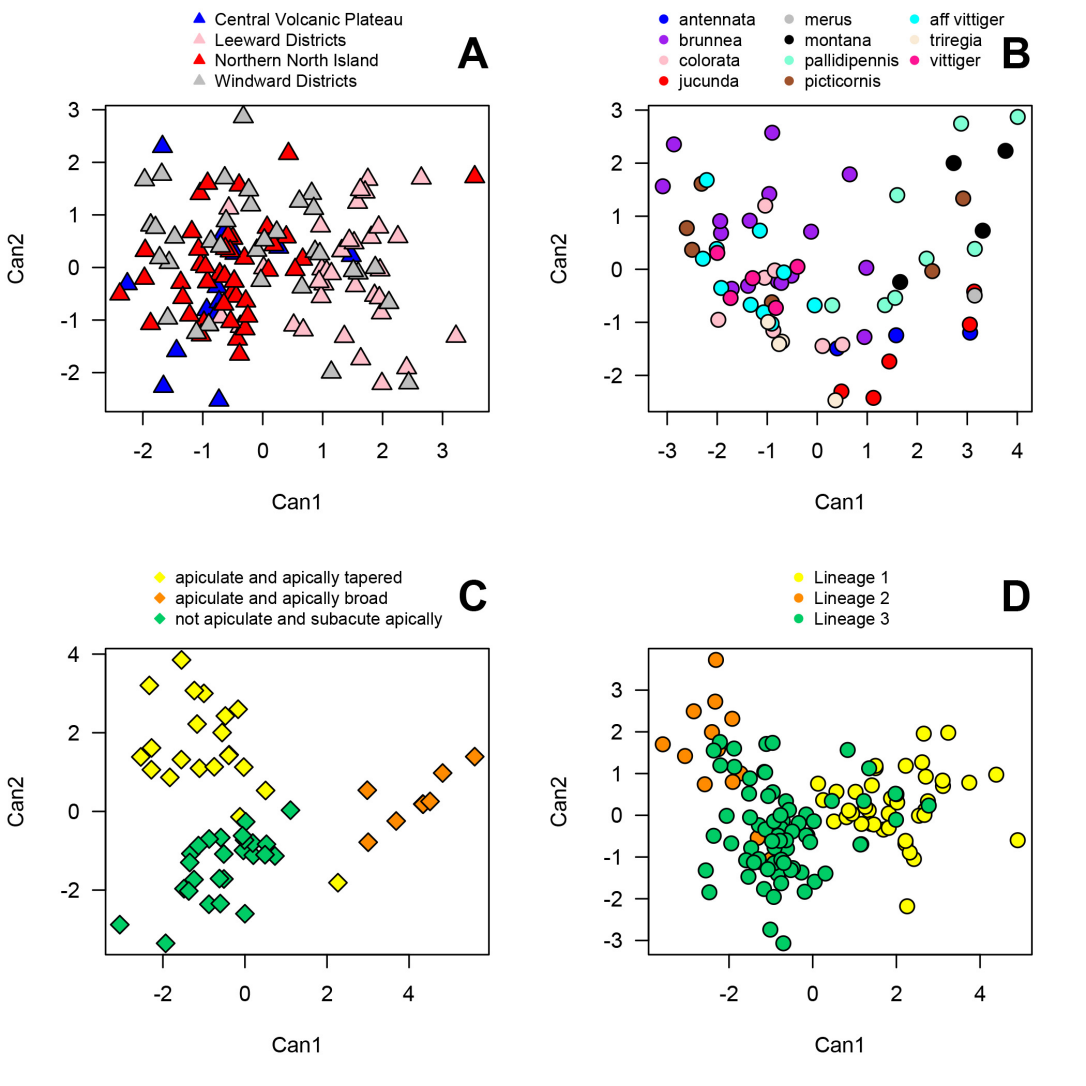
Canonical Discriminant analysis for *Eucolaspis* morphometric data. Can1 and Can2 are the first two canonical variables. (A) Morphometric relationships among *Eucolaspis* beetles collected from different ecological regions across New Zealand. (B) Morphometric relationships among identified voucher *Eucolaspis* specimens in NZAC collection: variation among the samples independently diagnosed to species. (C) Morphometric relationships among male *Eucolaspis* beetles with three different aedeagei types. (D) Morphometric relationships among the three mainland New Zealand genetic lineages of *Eucolaspis* (Lineages 1, 2 and 3).

Punctures on pronotum (PPD) and head (HPD) were the two characters contributing to the separation among beetles of different ecological regions of New Zealand. Beetles from Northern North Island and Central Volcanic Plateau were morphometrically similar to each other (*p* = .197), while Leeward districts samples were morphologically distant from other regions (*p* < .001) (Fig 3A). Canonical variable 1 (Can1) explained about 91% of variation among the ecological regions (Fig 3A). PPD contributed most to the Can1, while HPD contributed most to canoncial variable 2 (Can2). PPD, pronotum length (PL), body length (BL) and anterior elytral puncture density (AEPD) were the characters that significantly separated beetles from different host plants. Samples from apple and blackberry appeared to cluster together, while samples from manuka were very diverse with no clustering. PPD contributed more to the separation than the other three characters (*F* (7, 123) = 12.3, *p* < .001). Can1 and Can2 together explained about 89% variation among the beetles from different host plants.

#### Museum samples

Morphometric analysis of identified specimens in museum collections showed no distinct clusters. Overlap of data from different species, such as *E*. *vittiger*, *E*. *colorata* and *E*. *brunnea* suggested poor phenotypic separation of current species (Fig 3B). Individuals assigned to *E*. *picticornis* were morphologically highly variable and did not cluster together. The four *E*. *montana* paratypes from Broun’s collection varied considerably, highlighting instability of the existing taxonomy (Fig 3B). Among the ten morphological characters measured, only elytral width (EW), puncture density on head (HPD) and posterior elytral region (PEPD) contributed to significant variation among “species”. PEPD contributed the most variation (*F* (10, 58) = 11.77, *p* < .001). Can1 explained about 73% of variation among “species”, whereas Can2 explained about 19% of variation (Fig 3B).

### Genetic, ecological and geographic associations

**Fig 4:**
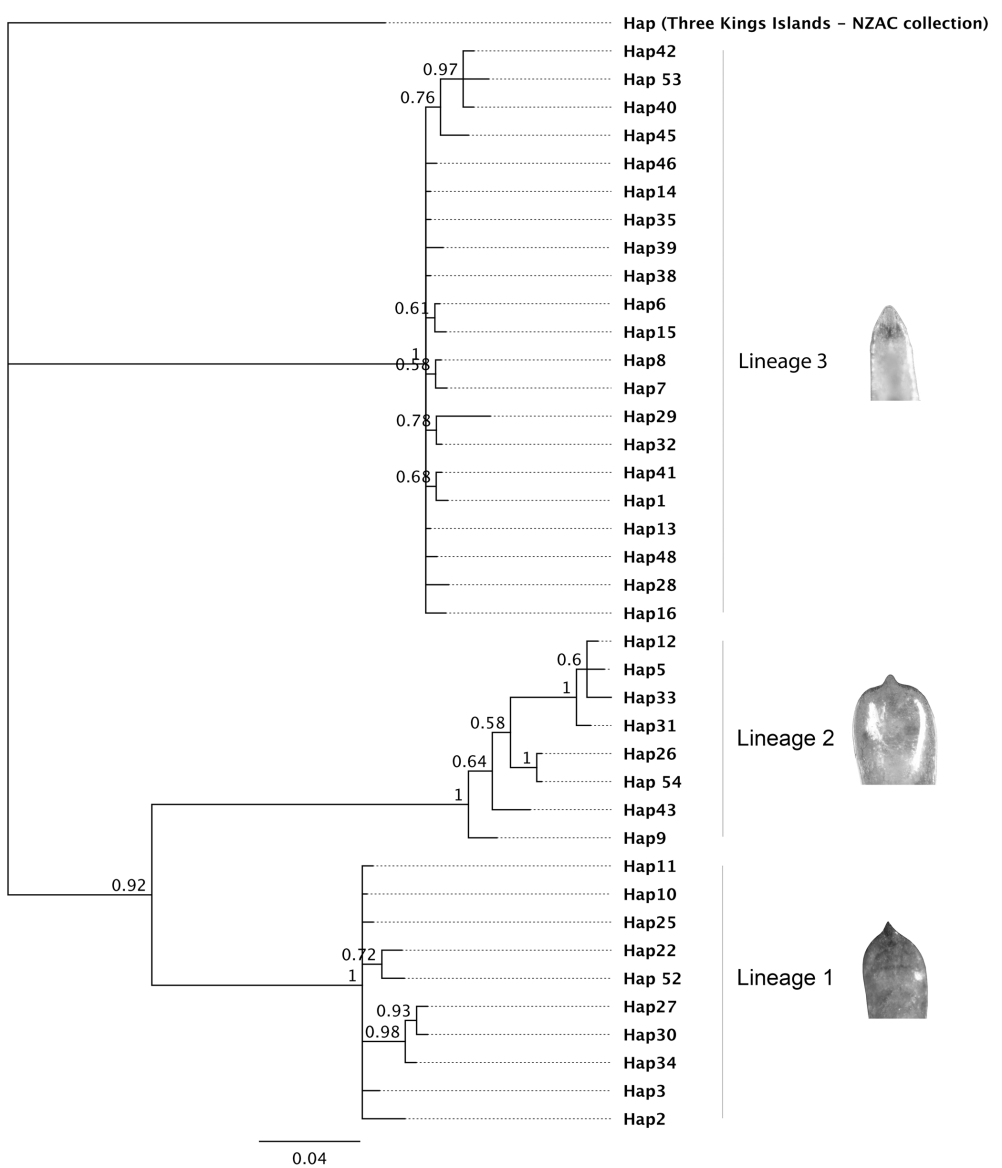
Bayesian phylogeny of New Zealand *Eucolaspis* (COI region of mtDNA) using GTR+G+I model. SBL = 0.924967. Node labels indicate posterior probabilities, and tip labels indicate corresponding taxon. Images of male genitalic aedeagus tip shape corresponding to each lineage are shown.

The 117 aligned mtDNA COI sequences (617 bp) from mainland New Zealand *Eucolaspis* comprised 39 haplotypes, with an additional haplotype identified from Three Kings Islands specimens. Haplotype diversity (Hd ± S.D.) was 0.97 ± 0.01, and nucleotide diversity per site (Π ± S.D.) was 0.0634 ± 0.0046. The alignment contained 129 variable positions and 97 parsimony informative sites. Reconstructed phylogeny of these haplotypes, using Three Kings Islands haplotype (HapTK-NZAC) as an outgroup, showed three well-supported lineages in the mainland ingroup (Lineage 1, 2 and 3) (Fig 4). Phylogenetic inference using different methods (Minimum Evolution, Maximum Likelihood and Bayesian inference) yielded near identical topologies, with minor variation in placement of haplotypes within lineages. Species delimitation analysis using Geneious conducted on a Bayesian inference tree, confirmed the monophyly of the three lineages with the probability of correct identification of an unknown specimen by the sequence tree ranging from 0.87 to 0.98 (Table 2).

**Table 2:** Species delimitation analysis confirms monophyly of the thee mainland New Zealand lineages of *Eucolaspis*. Inter Dist closest = mean pairwise tree distance between the members of the focal species and members of the next closest species; P ID(strict) = mean probability of correctly identifying an unknown specimen of the focal species using placement on a tree sequence; Av (MRCA) = mean distance between the most recent common ancestor of a species and its members; P (randomly distinct) is the probability that a lineage has the observed degree of distinctiveness due to random coalescent processes. Input tree was constructed by Bayesian inference method using GTR+G+I model.

*“Species”*	*Closest “species”*	*Intra Dist*	*Inter Dist closest*	*P ID (strict) (95% CI)*	*Av (MRCA-tips)*
Lineage 1	2	0.025	0.260	0.93 (0.84, 1.0)	0.0138
Lineage 2	1	0.041	0.260	0.87 (0.77, 1.0)	0.0378
Lineage 3	1	0.018	0.328	0.98 (0.92, 1.0)	0.0092

**Fig 5:**
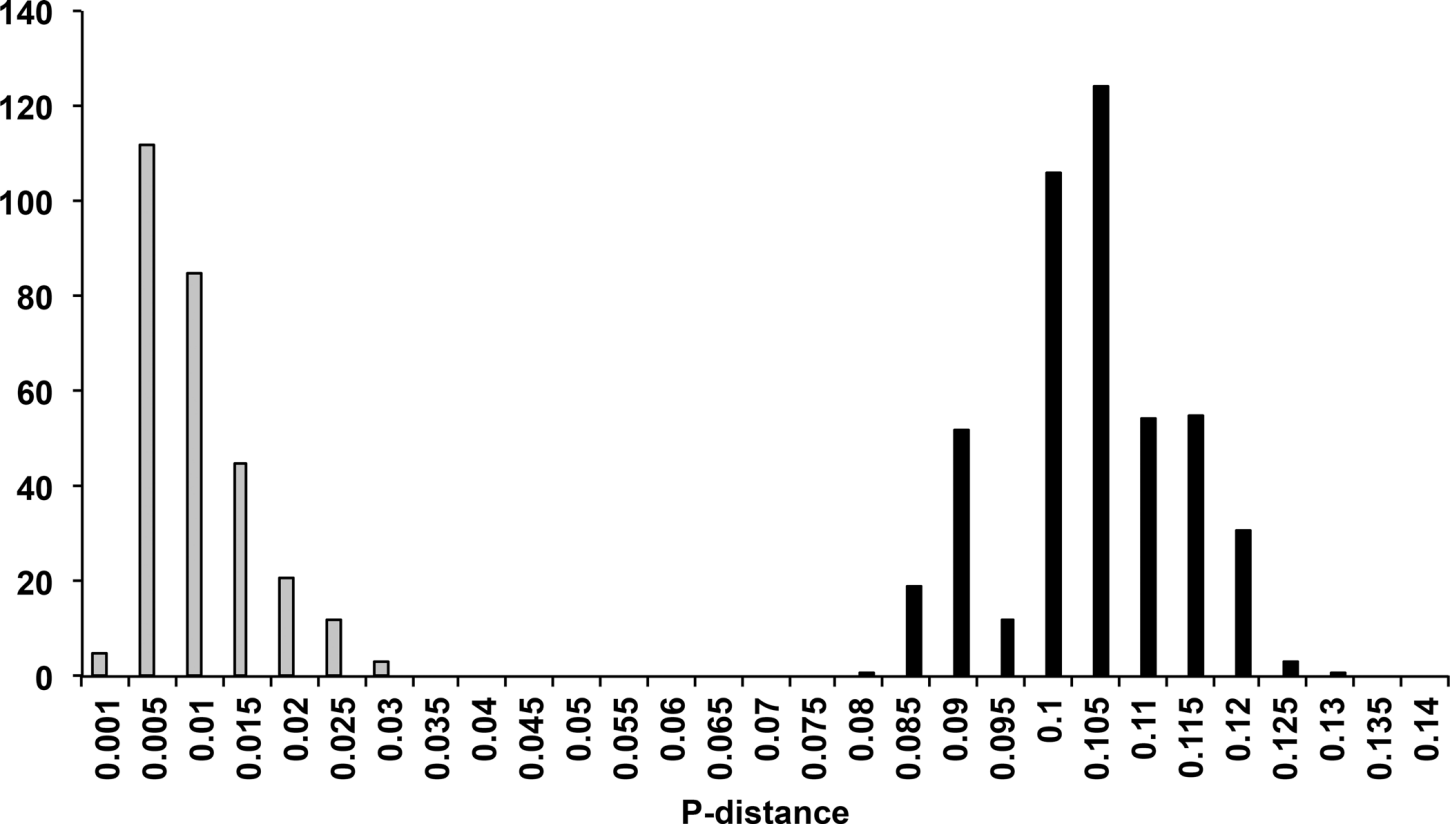
Frequency distribution of pairwise genetic distances (P-distances) among New Zealand *Eucolaspis* haplotypes. P-distances are calculated as number of nucleotide base differences per site between sequences. Grey bars represent pairwise intra-lineage distances whereas black bars represent pairwise inter-lineage distances.

The overall, mean genetic distance (p-distance ± standard error) among haplotypes was 0.068 ± 0.006 (measured using MEGA6). Lineage 2 had the highest within-group mean genetic distance (0.018 ± 0.004) compared to the other two lineages (Lineage 1: 0.012 ± 0.002; Lineage 3: 0.007 ± 0.002). Intra-lineage pairwise genetic distances ranged from 0.1% to 3% whereas inter-lineage pairwise genetic distances ranged from 8% to 12.7% (Fig 5). Mean net genetic distance (P-distance) measured as sequence divergence between lineages varied from 7.3% (lineages 1 and 2) to 10% (lineages 1 and 3) (Table 3). Lineages 1 and 2 were genetically more similar to each other than to Lineage 3 at this locus (Table 2). Phylogenetic analysis using entire CO1 fragment (~1400 bp) for respresentative individuals from these three lineages also conferred similar evolutionary relationships among the lineages (S1 Fig).

**Table 3:** Estimates of net evolutionary divergence between groups of *Eucolaspis* mtDNA (COI) sequences. The number of base substituions per site from estimation of net average between groups of sequences are shown. P-distances (lower triangular half of the table) and Maximum Likelihood (ML) distances (upper triangular half of the table) are shown. Standard errors were calculated through bootsrap procedures (500 replicates). ML analyses were conducted using the Tamura 3-parameter (T92) model [37]. The rate variation among sites was modeled with a gamma distribution (shape parameter = 0.15). Evolutionary analyses were conducted in MEGA6 [31].

Lineage	P-distance ± S.E. (lower) and ML distance* ± S.E. (upper)		
	1	2	3
1	-	0.143 ± 0.030	0.203 ± 0.045
2	0.073 ± 0.010	-	0.229 ± 0.048
3	0.095 ± 0.011	0.100 ± 0.010	-

*using T92+G model.

**Fig 6:**
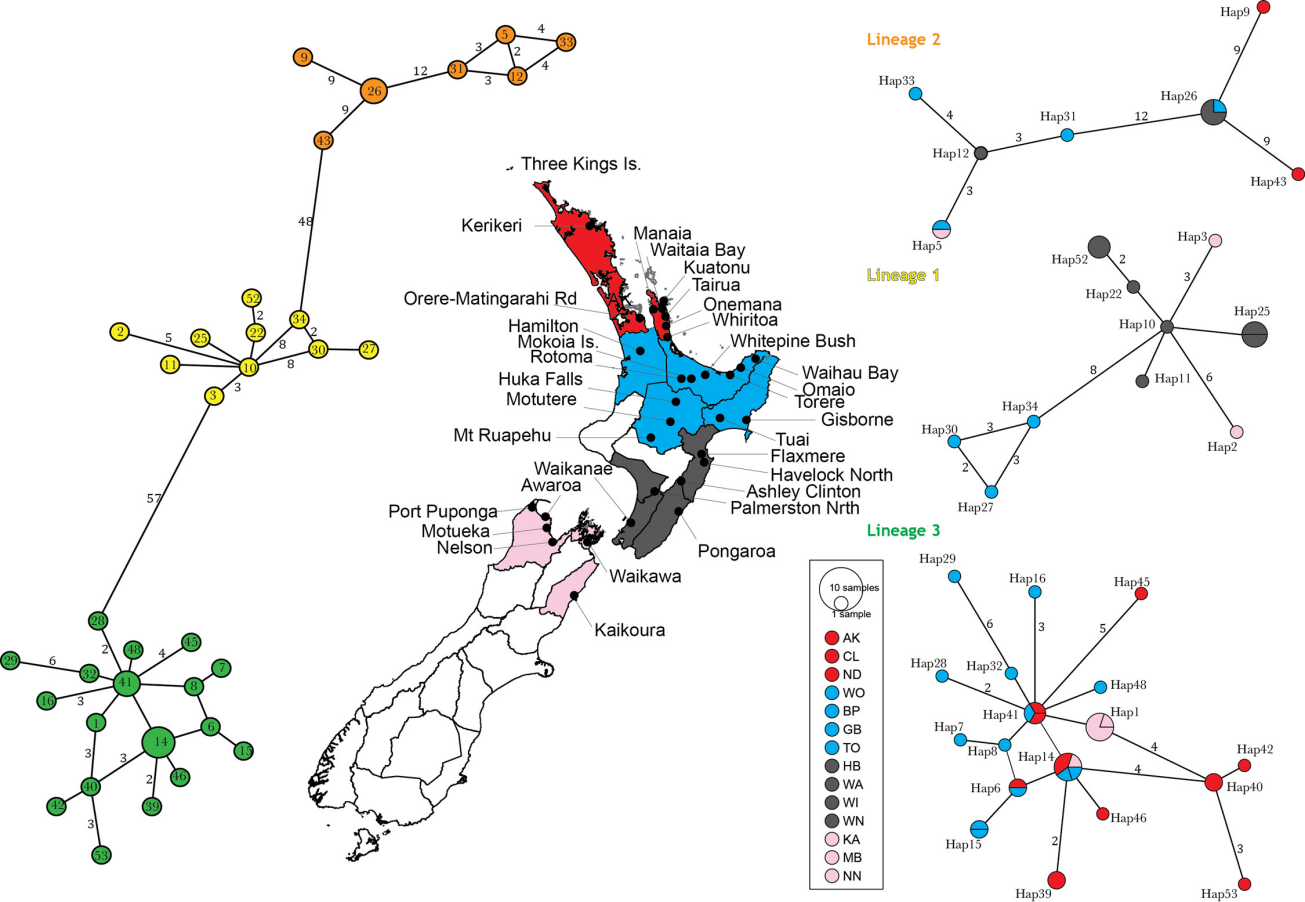
Geographical distribution of *Eucolaspis* lineages in mainland New Zealand. Total haplotype network structure (left) comprises three main lineages; yellow: Lineage1, orange: Lineage 2, green: Lineage 3. Dark circles on the map represent sampling sites with the distribution of haplotypes of each lineage (right) in broad regions. Regional entomological codes [38] are: AK-Auckland, ND-Northland, CL- Coramandel, BP-Bay of Plenty, GB-Gisborne, TO- Taupo, WO-Waikato, HB- Hawkes Bay, WA- Wairarapa, WI- Wanganui, WI Wellington, Pink: MB- Marlborough, NN- Nelson, KK- Kaiukoura. Size of the node (networks on the right) denotes number of haplotypes found.

The spatial distribution of the three *Eucolaspis* lineages showed clear demarcation between East and West, with lineage 1 occupying mostly Eastern areas (Fig 6). Where multiple beetle specimens were available we found that two lineages were sometimes represented at the same locality, whereas one locality (Torere—Bay Of Plenty) had representatives of all three lineages (n = 5). Lineage 2, which had more within-lineage genetic diversity than the other two, had wide geographic distribution and occured frequently in sympatry with one or both lineages 1 and 3. Among the host plants sampled in mainland New Zealand, blackberry (*Rubus fruticosus*) and manuka (*Leptospermum scoparium*)were used by all three *Eucolaspis* lineages, apple (*Malus domesticus*) was used only by lineages 1 and 2, and kanuka (*Kunzea ericoides*) was used by lineages 2 and 3.

### Male genitalia, morphology and molecular data

Three forms of male genitalic appendage or aedeagei, which differed primarily in the shape of the tip of aedeagus proper (type 1 –apiculate and tapered apically, type 2 –apiculate and broad apically, and type 3 –not apiculate and subacute apically) were found in a sample of 60 male beetles (Fig 4 and S2 Fig). Aedeagus type 3 was most different from the other two types, lacking a well-defined beak (tip). Individuals that belonged to mtDNA genetic lineage 1 possessed type 1 aedeagei, individuals of lineage 2 possessed type 2 and individuals of lineage 3 possessed type 3 aedeagei. An exception was in two males from a single locality in Nelson (collected on apples) that had type 1 aedeagei and belonged to lineage 3. Lineages 1 and 2 were genetically more similar to one another, and their aedeagei appeared to be relatively similar (Fig 4 and S2 Fig).

In morphometric analysis, elytra length (EL), pronotum length (PL) and pronotal punctures (PPD) were the only characters that differed among the males with different aedeagus types (Fig 3C). Can1 explained 71% variation, while Can2 explained 29% variation (Fig 3C). Males with aedeagus type 2 differed from the other two groups in having longer elytra and pronotum and lesser density of punctures on pronotum (squared Mahalanobis distance: between types 1 and 2 = 27.12, *p* < .001; between types 2 and 3 = 24.10, *p* < .001). Males with aedeagei type 1 and 3 mainly differed from one another in terms of puncture density on pronotum (squared Mahalanobis distance = 5.98, *p* < .001).

PPD, HPD, AEPD and EW differed significantly among beetles belonging to different haplotype lineages (Fig 3D). Can1 explained 86.4% variation among the lineages, while Can2 explained 13.6% (Fig 3D). Along Can1 (X-axis) PPD, HPD and AEPD were higher among indviduals of lineage 1, while EW was higher among individuals of lineages 2 and 3 (Fig 3D). PPD contributed most to the variation between lineages (*F* (2, 127) = 120.27; *p* < .001). Contrary to the genetic data, where lineages 1 and 2 were more similar (Tables 2 and 3), in morphometrics lineages 2 and 3 were more similar (squared Mahalanobis distance = 5.4; *p* < .001) than lineages 1 and 2 (squared Mahalanobis distance = 18.7; *p* < .001) (Fig 3D). Though canonical plots for haplotype lineages (Fig 3D) and aedeagus types (Fig 3C) appear different, it is important to note that these plots represent different datasets: Fig 3C includes data for males only, whereas Fig 3D includes data for both males and females. In both analyses, putative species (haplotype lineages, adedeagus types) were well separated from one another.

### Relationship with other Eumolpinae genera

**Fig 7:**
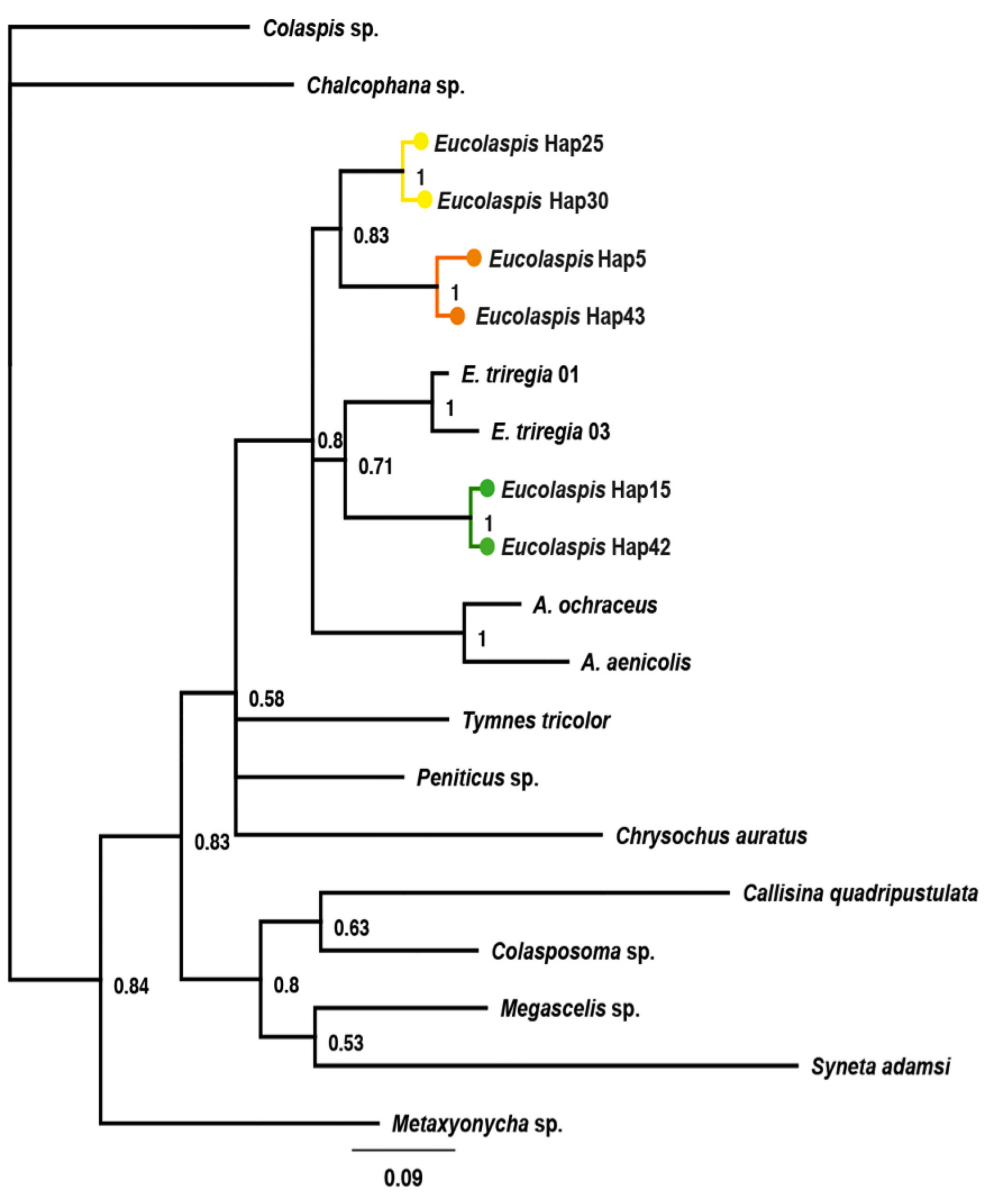
Bayeseian analyiss of mtDNA COI (~700 bp) from New Zealand and international Eumolpinae genera (Chrysomelidae) using a GTR+G+I model. SBL = 3.617. Node labels indicate posterior probability support, and tip labels indicate corresponding taxon. Mainland New Zealand *Eucolaspis* lineages are colour coded: Yellow- Lineage 1; Orange- Lineage 2; Green- Lineage 3.

In addition to our data, COI and 18S rDNA sequences corresponding to 9 different global Eumolpinae genera were obtained from GenBank. In the resulting mtDNA COI phylogeny constructed using Bayesian inference (Fig 7), *Eucolaspis* and *Atrichatus* formed a monophyletic lineage. New Zealand *Peniticus* was clearly more distant to *Eucolaspis* than *Atrichatus*, and indeed the current data yield a polytomy comprising *Eucolaspis* and *Atrichatus*. Other New Zealand genera in the subfamily Eumolpinae appear to be closely related to the *Eucolaspis* lineages. Analysis of rRNA 18S sequences constructed using the Maximum Likelihood criterion placed the New Zealand *Eucolaspis* lineages in a well-supported lineage along with another unidentified Eumolpinae taxon from New Caledonia (S3 Fig). This topology suggests that New Zealand *Eucolaspis* is loosely separated from other pacific Eumolpinae genera.

## Discussion

Mitochondrial DNA sequences, male genitalia and morphometric data provide strong evidence for just three mainland New Zealand species of *Eucolaspis* (lineages 1, 2 and 3) and a probably fourth on the Three Kings islands (HapTK-NZAC). Phylogenetic analysis showed well-supported lineages, sufficiently distinct to be consistent with different species. There was no support for a larger number of *Eucolapsis* species in mainland New Zealand.

The smallest inter-lineage genetic distance in *Eucolaspis* at this locus was about 8%, whereas mean interlineage genetic distance was about 10%; the inter-specific (8–12.7%) and intra-specific (0.1–3%) genetic distances did not overlap. There was a prominent gap between the intralineage and interlineage pairwise distances (Fig 6), indicating that taxonomic division is not being arbitrarily imposed on a continuous distribution of diversity. A similar gap was reported in *Crioceris* (Coleoptera: Chrysomelidae), where the maximum intraspecific genetic distance was about 2.5% and the interspecific distances ranged from 16.9 to 20.3% [39]. Similarly, in *Arsipoda* (Coleoptera: Chrysomelidae) the interspecific genetic distances varied from 8.1% to 14.4% while intraspecific genetic distances were much smaller (0.3–0.6%) [40]. Although this single locus evidence is insufficient on its own for taxonomic distinction [41,42], it is notable that just three mainland lineages are indicated rather than 15 [11–13] or 5 [15]. Analyses of phylogenetic relationships among New Zealand Eumolpinae genera suggest that the genera *Eucolaspis* Sharp and *Atrichatus* Sharp are more closely related to each other, than either is to *Peniticus* Sharp and this confirms the suggestions of Broun [11] and Shaw [15]. A fourth genus, *Pilacolaspis* Sharp, could not be included in this study as the only available specimens were old and did not yield amplifiable DNA.

Analysis of morphological characters from previously identified museum voucher specimens was not consistent with existing classification. Body size (length and width) of the beetles, the main characters that Broun [10–13] used in addition to body colour to describe many of his 13 *Eucolaspis* species, did not differ significantly among the randomly selected sample of different species named voucher specimens. Instead, other characters such as the width of elytra and puncture density on head and posterior elytra partitioned the species into clusters (Fig 3B). This provided a good impartial test of existing *Eucolaspis* taxonomy. Overlap of morphology of specimens supposedly representing different described species (such as *E*. *vittiger*, *E*. *colorata* and *E*. *brunnea*) supports in part the synonymy proposed by Shaw [15], although he based his inference on a different set of characters. Our examination of beetles from different genetic lineages in regards to the shape of the punctures, the main character Shaw (1957) used to delineate species, suggested that this character is highly inconsistent. The shape of the punctures varied among individuals within a population, and differences among individuals of different populations (and lineages) showed no consistency. However, puncture density on pronotum, head and elytra, characters also used by Shaw and Broun in species descriptions, displayed consistent differences among the genetic lineages.

Male genitalic shape and morphometric data coincide with genetic data, reiterating three mainland New Zealand lineages. Genitalic shape in males was consistent with a shared common ancestor of lineages 1 and 2. Variation in the shape of male genitalia also indicated that these reproductive structures are under evolutionary selection and this may reflect reproductive isolation, especially in sympatric populations. Reproductive isolation mechanisms such as variation in size and shape of cerci of male grasshoppers (*Parapodisma setouchiensis* and *P*. *subastris*) [43] and difference in cuticular hydrocarbon profiles that act as sex pheromones in leaf beetles *Chrysochus auratus* and *C*. *cobaltinus* [44] have been reported in sympatric populations. However, there is no information on reproductive isolation and / or incompatibility between *Eucolaspis* “species”.

Our results showed that only one lineage (putative species)–*Eucolaspis* lineage 1 infests apple orchards in Hawke’s Bay, New Zealand, while apples elsewhere in the country (e.g. Nelson) are infested by beetles of a different lineage. *Eucolaspis* feed on many different native and exotic plant species in New Zealand [20], and the wide range of host plants contributed to our sample of the three mainland New Zealand lineages suggests they are polyphagous and all could infest exotic fruit crops.

The three *Eucolapsis* lineages (putative species) were partitioned into North-West and South-East populations, and this was especially apparent in the Leeward Districts ecological region of New Zealand, which was occupied by lineage 1 (Fig 6). The Leeward districts, which is the driest region included in our sampling, are separated geographically from the rest of the country by the Ruahine and Tararua axial ranges in the North Island and the Southern Alps in the South Island. These ranges may act as a physical barrier, limiting mobility, however, Palmerston North (Windward districts) beetles were genetically similar to Hawke’s Bay (Leeward districts) populations, suggesting that contact and dispersal between regions is possible. We do not know if this dispersal is due to discontinuity in the ranges, or anthropogenic, or refelcts intermediate environmental conditions in this area. A similar East-West partitioning of distribution has been suggested in other New Zealand invertebrates including Onychophora [45], *Paryphanta* snails and corophiid amphipods (in [46]).

The genetic, genitalic and morphometric data utilized in our study complement each other but are not mutually exclusive, and therefore, integrative taxonomy is possible in this genus. Such an integration of different characters provides reliable taxonomic decisions [5], that reflect evolution [2]. Congruence of different types of data has been reported in many recent studies of Coleoptera (e.g., [47,48]). We conclude that there are only three putative species in mainland New Zealand unless others are very scarce or isolated. This is unlikely as our pattern of sampling encompassed the areas used to provide specimens for most of the earlier descriptions [10–13], which used material from just a few isolated locations. We also sampled through the North Island and in the Nelson-Marlborough and Canterbury regions of the South Island. *Eucolaspis* beetles are scarce or absent south of Canterbury region of New Zealand [49].

We therefore propose three mainland *Eucolaspis* taxa, distinguished by haplotype lineage, aedeagus shape, puncture density (on pronotum, head and anterior elytra) and elytra width. Beetles that belong to lineage 1 are distinguished morphologically by having denser puncturation (on pronotum, head and anterior elytra) and narrower elytra than the other two lineages. Given the available data, we propose the following names for *Eucolaspis* lineages as being appropriate: lineage 1 –*Eucolaspis puncticollis* (Broun 1880), based on resemblance of aedeagus tip shape with that described by Shaw [15]; lineage 2 –*E*. *picticornis* Broun 1893, based on comparison of 18S rDNA data with that of BMNH voucher 69636 GenBank accession DQ337133; lineage 3 –*E*. *jucunda* (Broun 1880), based on analogous aedeagus tip shape in Shaw [15] and congruence of 18S rDNA with that of BMNH voucher 696321 GenBank accession DQ337120.

Shaw [15] suggested *E*. *picticornis* as a junior synonym of *E*. *brunnea* (Fabricius, 1781), however, we feel that *E picticornis* is more appropriate to use in application to our data. Although *E*. *brunnea* (F., 1781) is the earliest name, we did not see the type material and cannot confirm that *E*. *brunnea* is consistent with the data we have gathered. In addition, there is a long-standing homonymy between New Zealand *E*. *brunnea* (originally described by Fabricius as *Chrysomela brunnea*, and later moved to *Colaspis* by White [9]), and North American “grape colaspis” *Colaspis brunnea* (Fabricuis, 1798) (originally described as *Galleruca brunnea*, and moved to *Colaspis* in 1801). To add more confusion, both species are horticultural pests; the North American grape colaspis has been sometimes referred to as the “bronzed beetle” (due to brown colour), and has been indexed in the American Review of Applied Entomology under an incorrect name of *Eucolaspis* (see discussion in Barber [50]). Although NZ *Eucolaspis brunnea* is the senior homonym, the name *Colaspis brunnea* is widely used for the North American species and the homonymy remains unresolved. The higher order evolutionary relationships of *Eucolaspis* (inter-generic and intra-subfamilial) within New Zealand, Pacific, and the world need to be investigated further.

## Supporting Information

S1 Fig Phylogenetic tree (for COI region of mtDNA) of New Zealand *Eucolaspis* constructed by Bayesian inference method using GTR+G model. SBL = 0.334443. Branch labels indicate substitutions per site, and tip labels indicate corresponding taxon. Lineages are highlighted with colour codes (Yellow = Lineage 1; Orange = Lineage2; Green = Lineage 3).
(TIF)

S2 Fig Three forms of aedeagus found among mainland New Zealand *Eucolaspis*. 1-apiculate and apically tapered, 2- apiculate and apically broad, 3- not apiculate and subacute apically.
(TIFF)

S3 Fig Bootstrap consensus tree of 18S rDNA sequence alignment of different genera in the subfamily Eumolpinae (Coleoptera: Chrysomelidae) constructed by Maximum Likelihood method using K2+G model. Log Likelihood = -4901.24; SBL = 0.25930817. Branch labels denote proportion (%) of tree occurrences in total of 500 replicates. *Eucolaspis* haplotypes from New Zealand (colour coded—Yellow = Lineage 1; Orange = Lineage 2; Green = Lineage 3) and one undescribed taxa (Eumolpinae sp.) from New Caledonia are highlighted (blue coloured branches).
(TIFF)

S1 Table A list of host plants of *Eucolaspis* in New Zealand [20]. (PDF)

S2 Table Details of fresh samples of *Eucolaspis* collected in New Zealand. (PDF)

S3 Table List of *Eucolaspis* species described by Fabricius (1781), White (1846), Broun (1880, 1893, 1903) and Shaw (1957). (PDF)

S4 Table GenBank (NCBI, USA) accession numbers (GI and Version) for global Eumolpinae taxa 18S rDNA sequences used in the current study. (PDF)
