# Supplementary figures and images for: Fifteen into Three Does Go: Morphology, Genetics and Genitalia Confirm Taxonomic Inflation of New Zealand Beetles (Chrysomelidae: Eucolaspis)
Source: PLoS One. 2015 Nov 23;10(11):e0143258. doi: 10.1371/journal.pone.0143258 (PMC4657921; doi:10.1371/journal.pone.0143258)

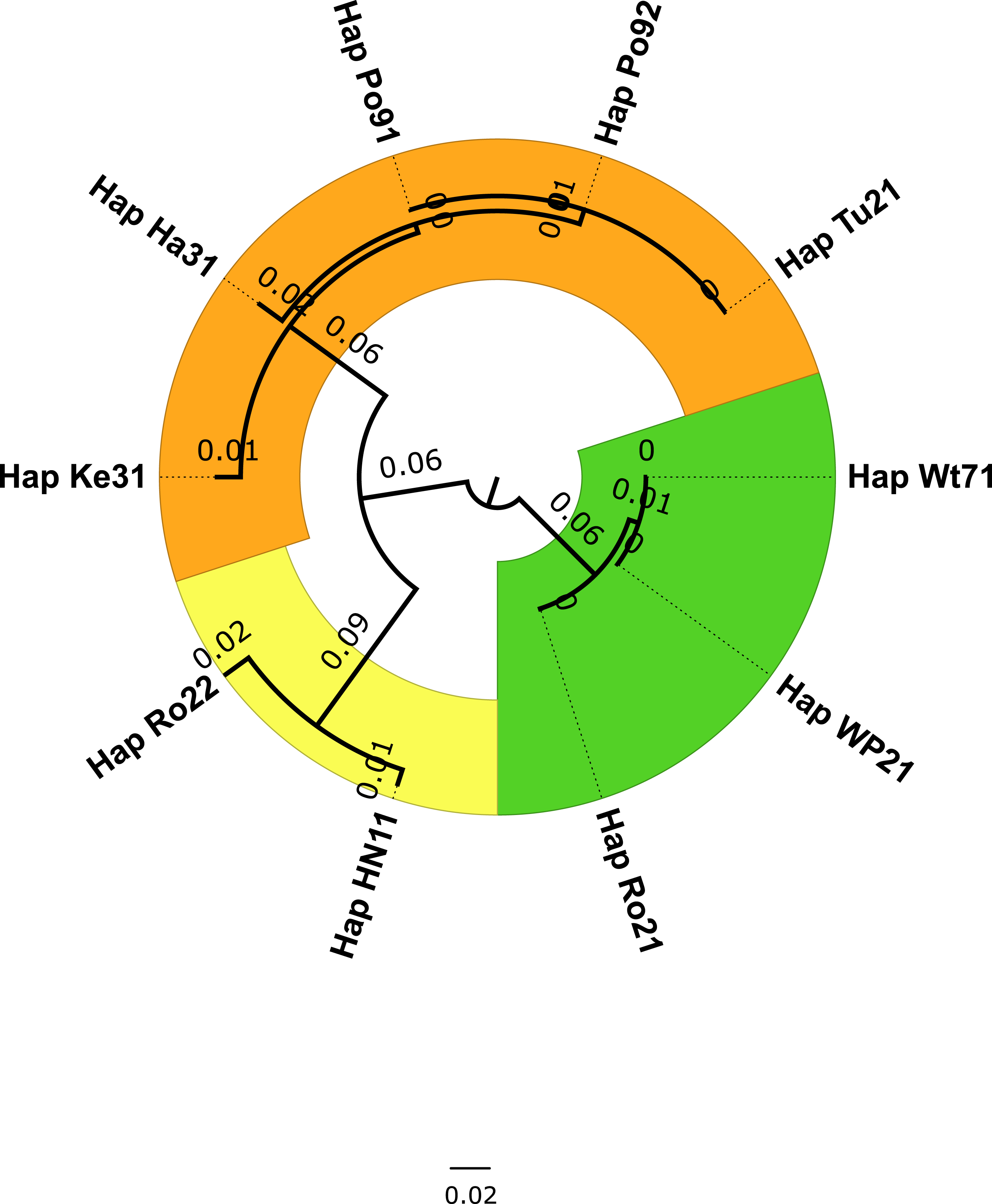

Supplement: S1 Fig — SBL = 0.334443. Branch labels indicate substitutions per site, and tip labels indicate corresponding taxon. Lineages are highlighted with colour codes (Yellow = Lineage 1; Orange = Lineage2; Green = Lineage 3). (TIF) [file pone.0143258.s001.tif]

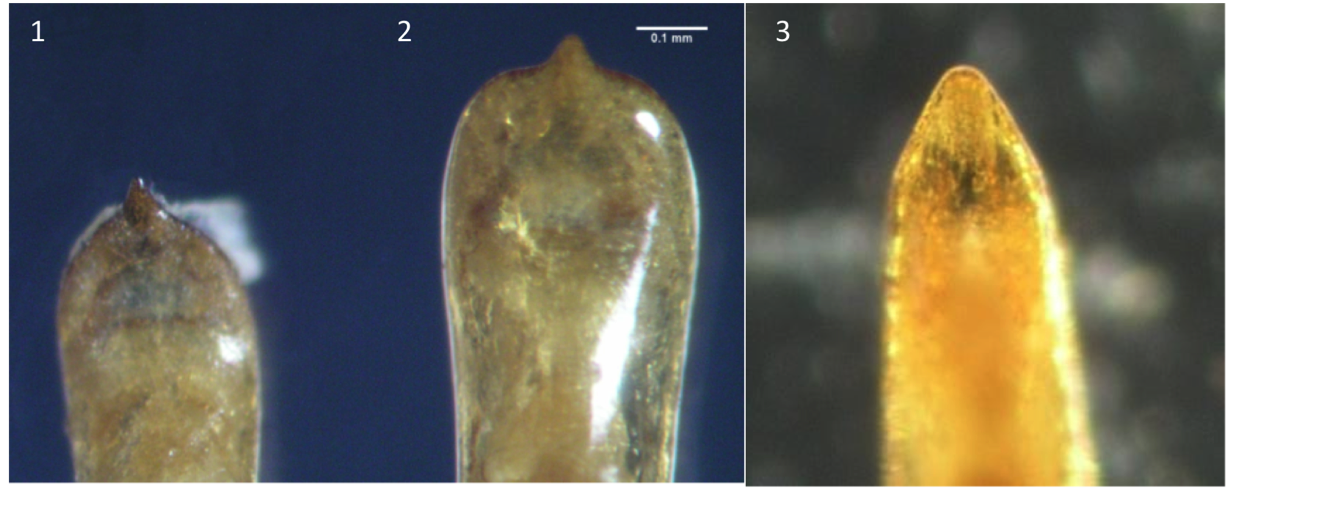

Supplement: S2 Fig — 1-apiculate and apically tapered, 2- apiculate and apically broad, 3- not apiculate and subacute apically. (TIFF) [file pone.0143258.s002.tiff]

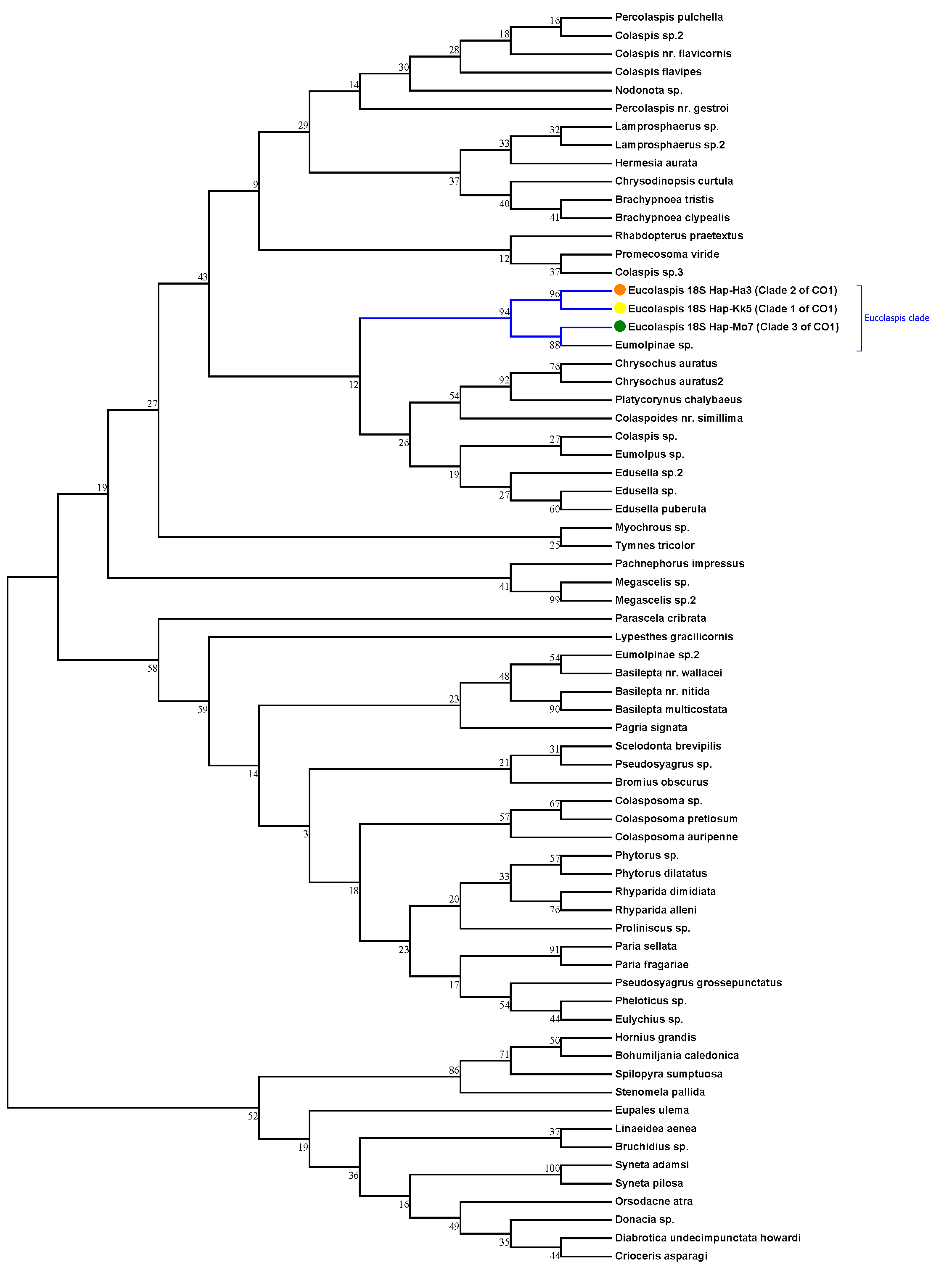

Supplement: S3 Fig — Log Likelihood = -4901.24; SBL = 0.25930817. Branch labels denote proportion (%) of tree occurrences in total of 500 replicates. Eucolaspis haplotypes from New Zealand (colour coded—Yellow = Lineage 1; Orange = Lineage 2; Green = Lineage 3) and one undescribed taxa (Eumolpinae sp.) from New Caledonia are highlighted (blue coloured branches). (TIFF) [file pone.0143258.s003.tiff]
