# Supplementary material for: Fifteen into Three Does Go: Morphology, Genetics and Genitalia Confirm Taxonomic Inflation of New Zealand Beetles (Chrysomelidae: Eucolaspis)
Source: PLoS One. 2015 Nov 23;10(11):e0143258. doi: 10.1371/journal.pone.0143258 (PMC4657921; doi:10.1371/journal.pone.0143258)
Supplement: S1 Table — (PDF) [file pone.0143258.s004.pdf]

Common Name	Scientific name	Family	Native / Introduced*	Reference
Pukanui	*Meryta sinclairii*	Araliaceae	Native	Coll. by R.E. Beever (NZAC specimen label)
Astelia	*Astelia* sp.	Asteliaceae	Native	NZAC specimen label
Boneseed	*Chrysanthemoides monilifera* ssp. *monilifera*	Asteraceae	Introduced	(1)
Californian thistle	*Cirsium arvense*	Asteraceae	Introduced	(2)
Alder	*Alnus glutinosa*	Betulaceae	Introduced	NZAC specimen label
Pink bindweed	*Calystegia sepium* ssp. *roseata*	Convolvulaceae	Native	(3)
Wineberry	*Aristotelia serrata*	Elaeocarpaceae	Native	M.A.Minor 2009, personal obs.
Prickly mingimingi	*Leptecophylla juniperina* subsp. *juniperina*	Ericaceae	Native	Personal obs., 2011
Blueberry	*Vaccinium corymbosum*	Ericaceae	Introduced	L.Hawes 2009, personal obs.
Acacia	*Acacia* sp.	Fabaceae	Introduced	(4)
NZ Broom	*Carmichaelia* sp.	Fabaceae	Native	Coll. by C.F. Butcher (NZAC specimen label)
Clover	*Trifolium* sp.	Fabaceae	Introduced	(4)
Gorse	*Ulex europaeus*	Fabaceae	Introduced	Coll. by P. Maddison (NZAC specimen label)
Geranium	*Geranium* sp.	Geraniaceae	Introduced	(4)
Black currant	*Ribes nigrum*	Grossulariaceae	Introduced	(5)
Gooseberry	*Ribes uva-crispa*	Grossulariaceae	Introduced	(5)
Fire weed	*Haloragis* sp.	Haloragaceae	Native	(3)
Tawa	*Beilschmiedia tawa*	Lauraceae	Native	Coll. by C.F. Butcher (NZAC specimen label)
Bay tree	*Laurus* sp.	Lauraceae	Introduced	(4)
Avocado	*Persea Americana*	Lauraceae	Introduced	(6)

Hangehange	*Geniostoma* sp.	Loganiaceae	Native	Coll. by P. Maddison (NZAC specimen label)
Hollyhock	*Alcea* sp.	Malvaceae	Introduced	(4)
Whau	Entelea arborescens	Malvaceae	Native	Coll. by J.C. Watt (NZAC specimen label)
Poor Knights houhere	Hoheria equitum	Malvaceae	Native	Coll. by J.S. Dugdale (NZAC specimen label)
Mallow	*Malva sylvestris*	Malvaceae	Introduced	(4)
Linden	*Tilia* sp.	Malvaceae	Introduced	S.Trewick 2008, personal obs.
Feijoa	*Acca sellowiana*	Myrtaceae	Introduced	Personal obs.,2009
Eucalyptus	*Eucalyptus* sp.	Myrtaceae	Introduced	(4)
Lilly pilly	*Syzygium smithii*	Myrtaceae	Introduced	NZAC specimen label
Manuka	*Leptospermum scoparium*	Myrtaceae	Native	(7, 8)
Kanuka	*Kunzea ericoides*	Myrtaceae	Native	Personal obs., 2010
Pohutukawa	*Metrosideros excelsa*	Myrtaceae	Native	(9)
Silver Beech	*Nothofagus menziesii*	Nothofagaceae	Native	NZAC specimen label
Kotukutuku	*Fuchsia excorticata*	Onagraceae	Native	(4)
Cymbidium	*Cymbidium* sp.	Orchidaceae	Introduced	(10)
Dendrobium	*Dendrobium* sp.	Orchidaceae	Introduced	(10)
Pine	*Pinus* sp.	Pinaceae	Introduced	(11)
Pittosporum	*Pittosporum* sp.	Pittosporaceae	Native	(4)
Kahikatea	*Dacrycarpus dacrydioides*	Podocarpaceae	Native	NZAC specimen label
Podocarpus	*Podocarpus* sp.	Podocarpaceae	Native	(4)
Totara	*Podocarpus totara* var. *totara*	Podocarpaceae	Native	S.Trewick 2011, personal obs.
Pohuehue	*Muehlenbeckia* sp.	Polygonaceae	Native	NZAC specimen label
Broad-leaved dock	*Rumex obtusifolius*	Polygonaceae	Introduced	Personal obs., 2007
Scarlet pimpernel	*Anagallis arvensis* ssp. *arvensis*	Primulaceae	Introduced	(12)
Weeping mapou	*Myrsine divaricata*	Primulaceae	Native	(4)

Red mapou	*Myrsine australis*	Primulaceae	Native	Coll. by J.C. Watt (NZAC specimen label)
NZ honeysuckle	*Knightia excelsa*	Proteaceae	Native	NZAC specimen label
Columbine	*Aquilegia* sp.	Ranunculaceae	Introduced	(2)
Hawthorn	*Crataegus*	Rosaceae	Introduced	(4)
Strawberry	*Fragaria* sp.	Rosaceae	Introduced	NZAC specimen label
Apple	*Malus domestica*	Rosaceae	Introduced	(5, 13)
Peach & nectarines	*Prunus persica*	Rosaceae	Introduced	(4)
Plum	*Prunus* sp.	Rosaceae	Introduced	(5)
Cherry	*Prunus* sp.	Rosaceae	Introduced	(4)
Apricot	*Prunus armeniaca*	Rosaceae	Introduced	NZAC specimen label
Pear	*Pyrus communis*	Rosaceae	Introduced	(5)
Rose	*Rosa indica*	Rosaceae	Introduced	(4)
Blackberry	*Rubus fruticosus*	Rosaceae	Introduced	(5)
Raspberry	*Rubus idaeus*	Rosaceae	Introduced	(5)
Coprosma	*Coprosma* sp.	Rubiaceae	Native	(4)
Poplar	*Populus* sp.	Salicaceae	Introduced	(Lysaght 1930)
Titioki / NZ Ash	*Alectryon excelsum*	Sapindaceae	Native	(4)
Camellia	*Camellia* sp.	Theaceae	Introduced	(14)
Elm	*Umulus* sp.	Ulmaceae	Introduced	(4)
Mahoe	*Melicytus ramiflorus*	Violaceae	Native	Coll. by R.C. Craw (NZAC specimen label)
Violet	*Viola* sp.	Violaceae	Introduced	(4)
Grape	*Vitis* sp.	Vitaceae	Introduced	(15)

***Status verified from http://www.NZPCN.org.nz (16)**

References:

1. Winks CJ, Fowler SV, Smith LA. Invertebrate fauna of boneseed, *Chrysanthemoides monilifera* ssp. *monilifera* (L.) T. Norl. (Asteraceae: Calenduleae), an invasive weed in New Zealand. N Z Entomol. 2004;27:61-72.
2. Rogers DJ, Cole LM, Fraser TM, Walker JTS, Brownbridge M. Management of bronze beetle in organic production - 2008. The Horticulture and Food Research Institute of New Zealand, 2008.
3. Kuschel G. Beetles in a suburban environment: a New Zealand case study. The identity and status of Coleoptera in the natural and modified habitats of Lynfield, Auckland (1974-1989). Auckland: DSIR Plant Protection Report 3, 1990.
4. Lysaght AM. Bronze beetle research. New Zealand Department of Scientific and Industrial Research, Wellington, New Zealand. 1930;Bulletin 25:32 pp.
5. Miller D. The Bronze beetle, its habits and control as an orchard pest. N Z J Agric. 1926;32(1):9-14.
6. New Zealand Avocado Growers' Association & Industry Council Ltd. Avocado: Avogreen Insect Biology 2011 [08/02/2012]. Available from: http://www.nzavocado.co.nz/index.php/pi_pageid/86.
7. Broun T. Manual of the New Zealand Coleoptera: The New Zealand Institute, Wellington; 1893. 1303-6 p.
8. White A. Insects. In: Richardson J, Gray JE, editors. The Zoology of the Voyage of HMS Erebus &Terror. 2. London1846. p. 23.
9. Martin NA. Pohutakawa leaf miner - *Neomycta rubida* 2010 [updated 29/04/201008/02/2012]. Available from: http://nzacfactsheets.landcareresearch.co.nz/factsheet/OrganismProfile/Pohutukawa_leaf_miner_-_Neomycta_rubida.html.
10. Dymock JJ, Holder PW. Nationwide survey of arthropods and molluscs on cut flowers in New Zealand. N Z J Crop Hortic Sci. 1996;24(3):249-57.
11. Kay MK. Bronze beetle. Rotorua: New Zealand Forest Service, Rotorua, New Zealand, 1980.
12. Auckland Botanic Gardens. Pohutakawas and related species for Auckland gardens. 1999.
13. Huntley RH, editor Remarks on some of the coleopterous insects which injure fruit and other trees in the neighbourhood of Wellington. Wellington Philosophical Society; 1867; Wellington, New Zealand.
14. Royal New Zealand Institute of Horticulture. Bug detection 2011 [17/08/2011]. Available from: http://www.rnzih.org.nz/pages/Bug_Detection.htm.
15. Woodfin JC. Control of vine diseases and pests occurring in New Zealand. N Z J Agric. 1927;35:298-309.
16. NZPCN. New Zealand Plant Conservation Network 2012 [29/06/2012]. Available from: http://www.nzpcn.org.nz/flora_search.asp?scfSubmit=1.
