# Supplementary material for: Fifteen into Three Does Go: Morphology, Genetics and Genitalia Confirm Taxonomic Inflation of New Zealand Beetles (Chrysomelidae: Eucolaspis)
Source: PLoS One. 2015 Nov 23;10(11):e0143258. doi: 10.1371/journal.pone.0143258 (PMC4657921; doi:10.1371/journal.pone.0143258)
Supplement: S2 Table — (PDF) [file pone.0143258.s005.pdf]

Collection locality	Collection year	Host plant	New Zealand Ecological region*	Sample code	Latitude (S)	Longitude (E)	Haplotypes present
**Ashley Clinton**	2011	Blackberry	Leeward Districts	AC2	-39.932736	176.299615	Hap 25
**Awaroa**	2009	Manuka	Windward Districts	Aw5	-40.85589	172.995186	Hap1
**Gisborne**	2011	Manuka	Leeward Districts	Gi5	-38.856333	177.903722	Hap 48
**Motueka**	2011	Manuka	Windward Districts	Go5	-41.116667	173	Hap5, Hap1
**Motueka**	2009	Wineberry	Windward Districts	Go10	-41.016667	173.016667	Hap1
**Hamilton**	2009	Blueberry	Northern North Island	Ha3	-37.783333	175.266667	Hap12, Hap13, Hap14, Hap15, Hap16
**Flaxmere**	2009	Apple	Leeward Districts	Fl1	-39.638678	176.790833	Hap22

**Havelock North**	2009	Apple	Leeward Districts	HN1	-39.627342	176.892972	Hap52,25
**Havelock North**	2010	Linden	Leeward Districts	HN4	-39.667851	176.888671	Hap52
**Havelock North**	2010	Manuka	Leeward Districts	HN5	-39.667851	176.888671	Hap52
**Huka Falls**	2011	Kanuka	Central volcanic plateau	Hu7	-38.649363	176.089768	Hap15
**Kaikoura**	2010	Manuka	Leeward Districts	Kk5	-42.4	173.666667	Hap3, Hap2
**Kerikeri**	2009	Blueberry	Northern North Island	Ke3	-35.216667	173.933333	Hap9
**Kuaotunu**	2011	Mixed[+]	Northern North Island	Ku8	-36.754744	175.7278293	Hap41, Hap42
**Kuaotunu**	2011	Manuka	Northern North Island	Ku5	-36.754744	175.7278293	Hap53
**Manaia**	2011	Manuka	Northern North Island	Ma5	-36.853724	175.452517	Hap40, Hap44, Hap45

**Mokoia Island**	2009	Blackberry	Central volcanic plateau	MI2	-38.066667	176.283333	Hap5
**Motutere**	2011	Kanuka	Central volcanic plateau	Mo7	-38.8938408	175.950552	Hap41
**Mt Ruapehu**	2009	Manuka	Central volcanic plateau	MR5	-39.183333	175.55	Hap6, Hap7, Hap8
**Nelson**	2009	Apple	Windward Districts	Ne1	-41.308374	173.121185	Hap14
**Omaio**	2011	Manuka	Northern North Island	Om5	-37.8484122	177.5887357	Hap29
**Orere**	2011	Kanuka	Northern North Island	O-M7	-36.9959616	175.2679749	Hap46
**Palmerston North**	2009	Apple	Windward Districts	PN1	-40.369705	175.601574	Hap25
**Palmerston North**	2009	Blackberry	Windward Districts	PN2	-40.413202	175.662503	Hap25
**Pongaroa**	2012	Totara	Leeward	Po9	-40.538818	176.196596	Hap54, Hap26

			Districts				
**Pongaroa**	2012	Kanuka	Leeward Districts	Po7	-40.538818	176.196596	Hap54
**Port Puponga**	2010	Manuka	Windward Districts	PP5	-40.566667	172.6	Hap1
**Rotoma**	2011	Blackberry	Central volcanic plateau	Ro2	-38.0571129	176.6438101	Hap34, Hap35
**Onemana**	2011	Mixed[+]	Northern North Island	On8	-37.1721595	175.8498838	Hap38
**Tairua**	2011	Kanuka	Northern North Island	Ta7	-36.9774049	175.8391513	Hap38, Hap39, Hap40
**Torere**	2011	Mixed[+]	Northern North Island	To8	-37.9186089	177.5039606	Hap30, Hap31, Hap32
**Tuai**	2011	Blackberry	Axial Ranges	Tu2	-38.659778	177.06665	Hap26, Hap27
**Waihau Bay**	2011	Kanuka	Northern North Island	Wa7	-37.6598527	177.8175447	Hap28

**Waikanae**	2009	Blackberry	Leeward Districts	Wk2	-40.866667	175.066667	Hap10, Hap11
**Waikawa**	2010	Manuka	Windward Districts	Ww5	-41.2905398	174.0401655	Hap1
**Waitaia Bay**	2011	Kanuka	Northern North Island	Wt7	-36.774092	175.72134	Hap39
**Whiritoa**	2011	Manuka	Northern North Island	Wh5	-37.3149303	175.882591	Hap6
**White Pine bush**	2011	Blackberry	Central volcanic plateau	WP2	-38.0129026	176.9480455	Hap33

*Eco-regions assigned following (1)

+Mixed vegetation of manuka, kanuka and blackberry

References:

1. McGlone M. Ecoregions - Te Ara Encyclopedia of New Zealand, updated 25-Sep-11 2011 [5/01/2012]. Available from: http://www.teara.govt.nz/en/ecoregions/1/1.
