# Supplementary material for: Fifteen into Three Does Go: Morphology, Genetics and Genitalia Confirm Taxonomic Inflation of New Zealand Beetles (Chrysomelidae: Eucolaspis)
Source: PLoS One. 2015 Nov 23;10(11):e0143258. doi: 10.1371/journal.pone.0143258 (PMC4657921; doi:10.1371/journal.pone.0143258)
Supplement: S3 Table — (PDF) [file pone.0143258.s006.pdf]

**Fabricius (1), White (2) & Broun (3-6) species**	**Shaw (7) species**	**Collection possibly holding types (holo or lecto types)**
Eucolaspis brunnea Fabricius (*Chrysomela brunnea*)	*E. brunnea*	BMNH
E. pallidipennis White (*Colaspis pallidipennis*)		
E. puncticollis Broun	*E. puncticollis*	BMNH
E. jucunda Broun		BMNH
E. subœnea Broun		BMNH
E. sculpta Broun		BMNH
E. mera Broun		BMNH
E. brevicollis Broun		BMNH
E. atrocerulea Broun		BMNH
E. ochracea Broun		BMNH
E. colorata Broun		BMNH
E. Montana Broun		BMNH
E. vittiger Broun		BMNH
E. picticornis Broun		BMNH
E. plicatus Broun		BMNH
	E. antennata	BMNH
	E. hudsoni	BMNH

1. Fabricius JC. Species Insectorum: Hamburgi et Kilonii; 1781. 552 p.
2. White A. Insects. In: Richardson J, Gray JE, editors. The Zoology of the Voyage of HMS Erebus &Terror. 2. London1846. p. 23.
3. Broun T. Manual of the New Zealand Coleoptera: The New Zealand Institute, Wellington; 1893. 1303-6 p.
4. Broun T. Manual of the New Zealand Coleoptera: The New Zealand Institute, Wellington; 1880. 621-6 p.
5. Broun T. XXXII.—Descriptions of new genera and species of New-Zealand Coleoptera. Journal of Natural History Series 8. 1909;4(21):275-91.
6. Broun T. LVI.—Descriptions of new Coleoptera from New Zealand. Journal of Natural History Series 6. 1893;12(71):374-92.
7. Shaw S. A revision of the New Zealand genera *Eucolaspis* Sharp and *Atrichatus* Sharp (Coleoptera: Chrysomelidae) with descriptions of two new species. Annals and magazine of natural history. 1957;12:641-55.
