# Supplementary material for: Fifteen into Three Does Go: Morphology, Genetics and Genitalia Confirm Taxonomic Inflation of New Zealand Beetles (Chrysomelidae: Eucolaspis)
Source: PLoS One. 2015 Nov 23;10(11):e0143258. doi: 10.1371/journal.pone.0143258 (PMC4657921; doi:10.1371/journal.pone.0143258)
Supplement: S4 Table — (PDF) [file pone.0143258.s007.pdf]

**Taxon**	**GI**	**VERSION**
Eucolaspis picticornis	85013420	DQ337133.1
Eucolaspis jucunda	85013407	DQ337120.1
Syneta adamsi	53689710	AY676694.1
Syneta pilosa	10946199	AF267464.1
Megascelis sp.	10946198	AF267463.1
Chrysochus auratus	10946197	AF267462.1
Colaspis sp.	10946196	AF267461.1
Eumolpus sp.	10946195	AF267460.1
Myochrous sp.	58577607	AJ781615.1
Edusella sp.	58577598	AJ781606.1
Edusella sp.2	58577597	AJ781605.1
Phytorus sp.	58577573	AJ781581.1
Megascelis sp.2	58577559	AJ781567.1
Lamprosphaerus sp.	58577588	AJ781596.1
Colaspis nr. *flavicornis*	58577583	AJ781591.1
Colasposoma sp.	58577579	AJ781587.1
Orsodacne atra	58577615	AJ781623.1
Donacia sp.	58577614	AJ781622.1
Crioceris asparagi	58577613	AJ781621.1
Bruchidius sp.	58577612	AJ781620.1
Linaeidea aenea	58577611	AJ781619.1
Diabrotica undecimpunctata howardi	58577610	AJ781618.1
Parascela cribrata	58577609	AJ781617.1
Pachnephorus impressus	58577608	AJ781616.1
Promecosoma viride	58577593	AJ781601.1
Percolaspis pulchella	58577592	AJ781600.1

Percolaspis nr. *gestroi*	58577591	AJ781599.1
Nodonota sp.	58577590	AJ781598.1
Lamprosphaerus sp.2	58577589	AJ781597.1
Hermesis aurata	58577587	AJ781595.1
Colaspis sp.2	58577586	AJ781594.1
Colaspis sp.3	58577585	AJ781593.1
Colaspis flavipes	58577584	AJ781592.1
Chrysodinopsis curtula	58577582	AJ781590.1
Brachypnoea tristis	58577581	AJ781589.1
Brachypnoea clypealis	58577580	AJ781588.1
Colasposoma pretiosum	58577578	AJ781586.1
Colasposoma auripenne	58577577	AJ781585.1
Proliniscus sp.	58577576	AJ781584.1
Pseudosyagrus sp.	58577575	AJ781583.1
Pseudosyagrus grossepunctatus	58577574	AJ781582.1
Phytorus dilatatus	58577572	AJ781580.1
Pheloticus sp.	58577571	AJ781579.1
Paria sellata	58577570	AJ781578.1
Paria fragariae	58577569	AJ781577.1
Eulychius sp.	58577567	AJ781575.1
Rhyparida dimidiata	58577566	AJ781574.1
Rhyparida alleni	58577565	AJ781573.1
Bromius obscurus	58577606	AJ781614.1
Lypesthes gracilicornis	58577605	AJ781613.1

Scelodonta brevipilis	58577604	AJ781612.1
Colaspoides nr. *simillima*	58577602	AJ781610.1
Platycorynus chalybaeus	58577601	AJ781609.1
Chrysochus auratus 2	58577600	AJ781608.1
Tymnes tricolor	58577599	AJ781607.1
Edusella puberula	58577596	AJ781604.1
Eumolpinae sp.	58577595	AJ781603.1
Rhabdopterus praetextus	58577594	AJ781602.1
Pagria signata	58577564	AJ781572.1
Eumolpinae sp.2	58577563	AJ781571.1
Basilepta nr. *wallacei*	58577562	AJ781570.1
Basilepta nr. *nitida*	58577561	AJ781569.1
Basilepta multicostata	58577560	AJ781568.1
Eupales ulema	58577557	AJ781565.1
Spilopyra sumptuosa	58577556	AJ781564.1
Bohumiljania caledonica	58577555	AJ781563.1
Stenomela pallida	58577554	AJ781562.1
Hornius grandis	58577553	AJ781561.1
